# Development of electrochemiluminescence immunochromatographic test strips for dengue virus

**DOI:** 10.3389/fbioe.2026.1881809

**Published:** 2026-08-27

**Authors:** Qinyi Zhang, Yao Qin, Xihan Wang, Liting Xiao, Yanshan Mai, Jie Wu, Hongbin Zhang, Ling Li

**Affiliations:** 1 School of Public Health, Guangdong Medical University, Zhanjiang, China; 2 Department of Basic Medical Research, General Hospital of Southern Theater Command, Guangzhou, China; 3 Zhuhai Center for Disease Control and Prevention, Zhuhai, China; 4 Guangdong Food and Drug Vocational College, Guangzhou, China

**Keywords:** denv, electrochemiluminescence, lateral flow immunochromatography, paper-based biosensor, point-of-care testing

## Abstract

**Introduction:**

Dengue virus (DENV) infection poses a serious threat to global public health. Rapid and sensitive point-of-care diagnostic methods are urgently needed for early detection and epidemic control.

**Methods:**

The dengue virus monoclonal antibody NS1-McAb1# was labeled as the labeled antibody and sprayed onto the binding pad using ruthenium tripyridine as the signal probe, and the dengue virus monoclonal antibody DN-REAB-G2-006 was fixed on the detection pad as the capture antibody. To mitigate potential interference from other substances in the sample, the sample pad and conjugate pad was pretreated with a solution containing heterophilic blocking reagent, and Elecsys buffer was selected as the optimal co-reactant. After dropping the sample onto the sample pad, chromatography and immunoreaction were performed sequentially. Then, cyclic voltammetry scanning was carried out in the test zone at a rate of 0.1 V/s for electrochemiluminescence (ECL) detection. A novel detection method was established by optimizing various parameters.

**Result:**

The ruthenium-labeled antibody was prepared by reacting Ru-NHS ester with NS1-McAb1# at a molar ratio of 50:1 for 10 h. Elecsys Buffer was selected as the co-reactant, with a sample-to-buffer volume ratio of 2:1. Whatman AE 100 membrane was used as the detection pad with a length set to 15 mm. The concentration ratio of labeled antibody to capture antibody was 1:2. The chromatographic time after sample loading was 10 min. Under these optimized conditions, the detection method achieved a minimum detection limit of 20.76 pg/mL, exhibited good specificity, with an intra-assay repeatability coefficient of variation of 4%, a storage stability coefficient of variation of 0.83%, and an inter-assay stability coefficient of variation of 0.81%. Clinical sample validation showed a *Kappa* value of 0.625 compared with nucleic acid testing results.

**Discussion:**

The developed ECLIA test strip provides a portable, rapid, and sensitive detection method for DENV, with potential applications in on-site testing across multiple scenarios. Although preliminary clinical evaluation showed acceptable concordance with nucleic acid testing, further large-scale validation using diverse clinical specimens is necessary to fully establish its diagnostic utility in real-world settings.

## 1 Introduction

Dengue fever is a self-limiting febrile illness caused by the DENV. It is an acute arboviral disease transmitted primarily through the bite of vector mosquitoes, notably *Aedes albopictus* and *Aedes aegypti*. The general population is universally susceptible to infection. Dengue fever is endemic across tropical and subtropical regions worldwide (Wei et al., 2019). Due to the impacts of climate change, the transmission of DENV in China has progressively expanded from the southern regions to the central and western areas, and even into temperate northern zones (Liu et al., 2020). The World Health Organization estimates that approximately 390 million dengue infections occur globally each year, among which about 96 million individuals exhibit clinical symptoms of varying severity (Rocklöv and Dubrow, 2020). Dengue fever is the mosquito-borne infectious disease with the highest incidence, the widest distribution, and the greatest economic burden, posing a serious threat to global public health security and economic development. In 2019, it was listed by the WHO as one of the top ten global health threats (Gao and Leng, 2024). DENV belongs to the genus Flavivirus within the family Flaviviridae. Based on serotypes identified following infection, it is classified into four distinct subtypes: DENV-1, DENV-2, DENV-3, and DENV-4. Following infection with a specific DENV subtype, the host develops long-term immunity against that homologous serotype; however, this confers no protective effect against other DENV subtypes. Subsequent infection with a heterologous DENV subtype increases the risk of severe disease, a phenomenon known as antibody-dependent enhancement (ADE) effect. Currently, research on DENV vaccines continues to advance globally. Although two DENV vaccines have been approved worldwide, neither has been authorized in China. Moreover, these two vaccines exhibit several limitations, including unclear criteria for dengue protection induced by vaccination, the risk of antibody-dependent enhancement post-vaccination, the inability of one vaccine to provide simultaneous protection against all four DENV serotypes, and restricted applicability to specific populations (Sun and Zheng, 2024). To prevent dengue fever outbreaks, rapid diagnosis serves as a crucial component of dengue prevention and control. Quickly and accurately identifying infected individuals enables early detection and treatment, as well as timely interruption of viral transmission, which can substantially reduce the risk of DENV spread and play a significant role in epidemic containment. Therefore, developing a point - of - care testing (POCT) method that is highly sensitive, accurate, and simple to operate holds important research significance for DENV surveillance.

Electrochemiluminescence detection technology does not require an external light source, eliminating the drawbacks of stray light and fluctuations caused by unstable light sources, thereby reducing noise and improving the signal-to-noise ratio (Fracassa et al., 2025). In addition, based on sensitive photoelectric detection technology, this method offers advantages such as high sensitivity, a wide linear range, and simple instrument configuration (Wang et al., 2023; Wang et al., 2025). To achieve quantitative analysis, the involvement of specific signal molecules is required as detection mediators. Ruthenium tris(bipyridine) [Ru(bpy)_3_^2+^] is an electrochemiluminescence-active substance with advantages such as high luminescence efficiency, repeatable excitation, high detection sensitivity, wide linear range, and good stability, making it suitable as a signal molecule for electrochemiluminescence detection. When ruthenium is reacted with a specific antibody under defined conditions, the resulting complex is referred to as the labeled antibody, which serves as the signal probe in electrochemiluminescence detection (Han et al., 2024).

Lateral flow immunochromatographic assay (LFIA) is a point-of-care testing technology that provides qualitative or semi-quantitative information (Li et al., 2023). The LFIA test strip consists of a sample pad, a conjugate pad, a detection pad, and an absorbent pad, with each component overlapping and embedded on a backing card using pressure-sensitive adhesive (Saweres-Argüelles et al., 2025). Taking the double-antibody sandwich method as an example, the principle involves spraying and immobilizing the capture antibody onto the detection pad, while the labeled antibody is sprayed and immobilized onto the conjugate pad (Hou et al., 2025). When the test sample is applied to the sample pad, capillary action drives the antigen in the sample to react with the labeled antibody on the conjugate pad in the first specific antigen-antibody reaction. The complex then migrates to the detection zone, where a second specific antigen-antibody reaction occurs, forming a double-antibody sandwich complex that can be detected. LFIA requires only the dilution of the test sample with a sample buffer and does not rely on specialized equipment or trained personnel for analysis, allowing for rapid and straightforward result interpretation. Additionally, this detection method has low production costs, is stable and reliable once established, and does not require special storage conditions. These advantages make LFIA an ideal choice for both laboratory testing and on-site detection (Ma et al., 2024).

The combination of ECL and LFIA constitutes electrochemiluminescence immunoassay (ECLIA), enabling the development of a novel quantitative POCT tool that operates on a paper-based platform, allowing for rapid quantitative detection of DENV. This method does not require large-scale instruments and is well-suited for on-site rapid testing in primary care settings such as remote areas.

**FIGURE 1:**
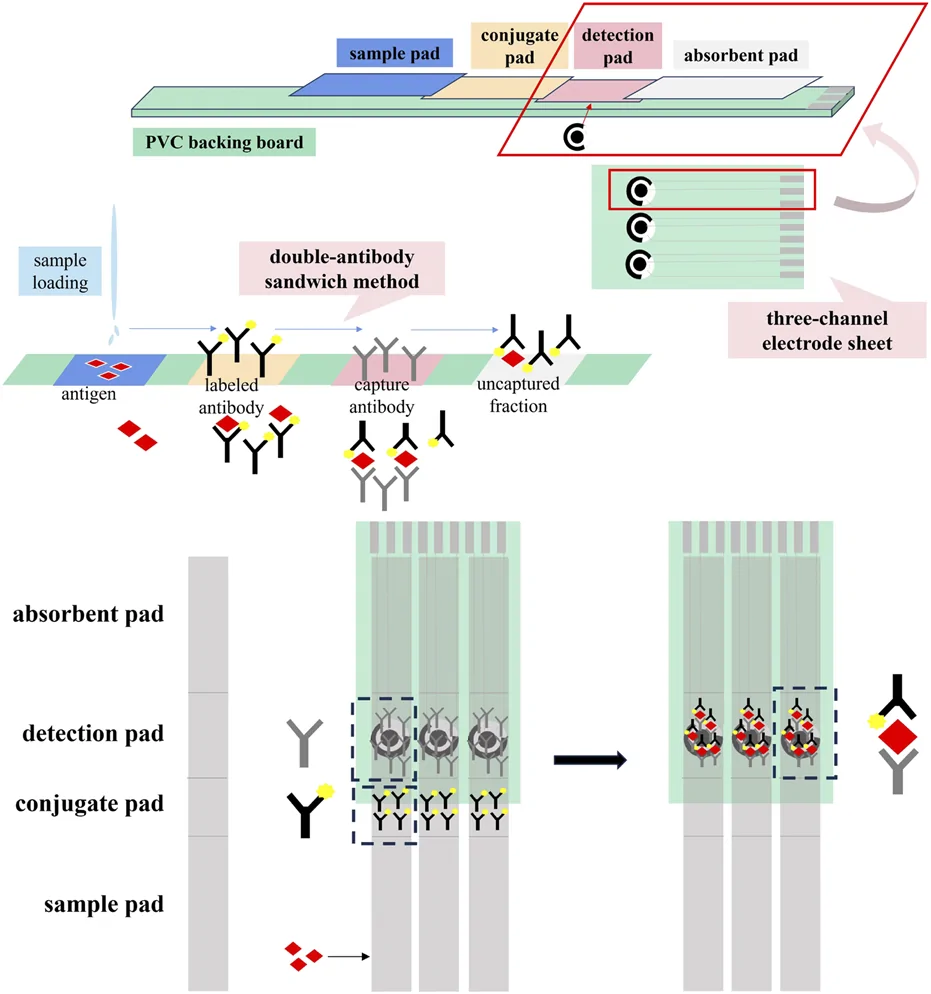
Schematic diagram of the electrochemiluminescence immunochromatographic test strip

As shown in Figure 1, the NS1 protein exhibits high stability and is one of the key proteins involved in the pathogenesis of DENV infection. NS1 is abundantly expressed in the early stages of DENV infection, and its levels are positively correlated with the severity of dengue fever, suggesting that early diagnosis of dengue can be achieved through the detection of the NS1 protein. Therefore, the NS1 protein was selected as the target for DENV antigen detection.

This study aims to establish a novel quantitative point-of-care testing platform that integrates electrochemiluminescence with lateral flow immunoassay technology on a paper-based substrate, enabling rapid quantitative detection of DENV and laying the foundation for the further development of portable kits and devices. This POCT innovation is designed to streamline the experimental process, improve detection speed and sensitivity, reduce costs, and expand applicability to a wider range of scenarios.

## 2 Materials and methods

### 2.1 Materials

#### 2.1.1 Reagents and consumables

DENV-NS1 antigen was purchased from Guangdong Biolight Biotechnology Co., Ltd. (China). The detection antibody NS1-McAb1# was obtained from Guangdong Biolight Biotechnology Co., Ltd. (China). The capture antibody DN-REAB-G2-006 was sourced from Guangdong Biolight Biotechnology Co., Ltd. (China). Ru-NHS ester [Ru(bpy)_2_(mcbpy-O-Su-ester) (PF_6_)_2_] was obtained from Shanghai Aladdin Biochemical Technology Co., Ltd. (China). Dimethyl sulfoxide (DMSO) was acquired from Shanghai Macklin Biochemical Technology Co., Ltd. (China). A 5 mL 7K MWCO Zeba™ spin desalting column was purchased from Thermo Fisher Scientific (United States). Elecsys buffer was sourced from Roche (Switzerland). Tripropylamine (TPA) was obtained from Sigma (United States). Glass fiber RB-45 was purchased from Huaiyuan Tongcheng Paper Products Co., Ltd. (China). The absorption pad ABP-S270 was obtained from Huaiyuan Tongcheng Paper Products Co., Ltd. (China). The nitrocellulose microporous membrane UniSart CN 95 was sourced from Sartorius (Germany). The nitrocellulose microporous membrane Whatman NO.1 was supplied by Whatman (United States). Whatman AE 100 was procured from Whatman (United States). The nitrocellulose microporous membrane PT-0.45 μm was purchased from Cytiva (United States). The three-channel electrode sheet was acquired from Guangzhou Weiguang Technology Co., Ltd. (China). 0.1 M PBS buffer (pH 8.5), 0.1 M PBS buffer (pH 7.4), and 0.01 M PBS buffer (pH 7.4) were prepared in-house.

#### 2.1.2 Instruments

A BPCL three-channel electrochemiluminescence detector was purchased from Guangzhou Weiguang Technology Co., Ltd. (China). A rotating disk mixer was obtained from Aicope International Co., Ltd. (United States). A Nanodrop 2000 ultra-micro spectrophotometer was acquired from Thermo Fisher Scientific (United States). An HGS510-1 benchtop gold sputtering machine was sourced from Hangzhou Feng Hang Technology Co., Ltd. (China). An HGS210 high-speed slitting machine was procured from Hangzhou Feng Hang Technology Co., Ltd. (China). A CTD300 numerically controlled strip cutting machine was supplied by Shanghai Jinbiao Biotechnology Co., Ltd. (China). A DHG-9240A forced air drying oven was provided by Shanghai Yiheng Scientific Instrument Co., Ltd. (China).

### 2.2 Methods

#### 2.2.1 Preparation of the ECL bioprobe

The NS1-McAb1# antibody, designated as the target for labeling, was conjugated with Ru-NHS ester. The resulting Ru-NHS ester-labeled NS1-McAb1# served as the electrochemiluminescence (ECL) bioprobe. The labeling procedure was performed in accordance with established protocols from relevant studies (Kitte et al., 2021; GUAN et al., 2016; Zhou et al., 2014).

The preparation method was as follows:

1. A 1 mg aliquot of Ru-NHS ester was removed from a −20 °C freezer and allowed to equilibrate to room temperature. Then, 10 μL of DMSO and 990 μL of 0.01 M PBS buffer (pH 7.4) were sequentially added, followed by vortex mixing until complete dissolution, to obtain a 1 mg/mL Ru-NHS ester solution.
2. A 1 mg aliquot of NS1-McAb1# was taken from the −20 °C freezer and equilibrated to room temperature. A 1 mL reaction system was designed, with a final concentration of NS1-McAb1# at 1 mg/mL, using 0.1 M PBS buffer (pH 8.5) as the coupling buffer. Ru-NHS ester solution and NS1-McAb1# were added at specific molar ratios (40:1, 50:1, 60:1, 80:1), and the volume was adjusted to 1 mL with 0.1 M PBS buffer.
3. After preparation, the reaction solution was wrapped in aluminum foil and placed on a rotating disk mixer, where it was allowed to react at room temperature at 35 r/min for a specified duration (4 h, 8 h, 10 h, 12 h).
4. After the thorough reaction of the ruthenium-antibody solution, a 5 mL 7 K MWCO Zeba™ spin desalting column was used to remove free Ru-NHS ester and other small-molecule impurities that did not bind to the antibody from the reaction mixture. Following the pretreatment of the spin desalting column, centrifugation was performed at 1,000 × g for 2 min. The eluate was collected under light-protected conditions, yielding the Ru-NHS ester-labeled NS1-McAb1#.

#### 2.2.2 Preparation of the immunochromatographic test strips

The immunochromatographic test strip is composed of a sample pad, a conjugate pad, a detection pad, and an absorbent pad.

First, a treatment solution was prepared using 0.01 M PBS (pH 7.4) to formulate 0.05 M Tris-HCl (pH 7.4), 1% BSA, 0.5% sodium caseinate, 0.5% trehalose, 2% sucrose, 0.5 mg/mL heterophilic blocking reagent (HBR), and 0.9% NaCl.

The sample pad was pretreated by uniformly spraying the treatment solution onto it, followed by drying at 37 °C for 12 h. After drying, the pad was cut into 7 mm × 20 mm strips for later use.

The conjugate pad was pretreated by evenly spraying the treatment solution onto the pad, followed by drying at 37 °C for 12 h. After drying, it was cut into strips with a width of 10 mm for later use.

The above Ru-NHS ester-labeled NS1-McAb1# was uniformly sprayed onto the aforementioned conjugate pad at a rate of 6 μl/cm using an HGS510-1 instrument, followed by drying at 37 °C for 2 h. After drying, the pad was cut into 7 mm × 10 mm strips for later use and stored protected from light.

The detection pad was cut into strips with a width of 20 mm for later use. The capture antibody DN-REAB-G2-006 was uniformly sprayed onto the detection pad at a rate of 6 μL/cm using an HGS510-1 instrument, followed by drying at 37 °C for 2 h. After drying, the pad was cut into strips measuring 7 mm × 20 mm for later use (the strip dimensions are subject to further adjustment).

The absorbent pad was cut into strips measuring 7 mm × 20 mm for later use.

The screen-printed electrode sheets were fixed onto a PVC backing board, and three sets of sample pads, conjugate pads, detection pads, and absorbent pads were overlapped and affixed onto the three channels of the electrode sheets, ensuring that the detection pads were positioned over the detection zones of the electrodes, to prepare the immunochromatographic test strips. This configuration allows for simultaneous detection of the sample, negative control, and positive control.

#### 2.2.3 The preparation of DENV-NS1 samples

DENV-NS1 antigen was taken out from a −20 °C freezer and equilibrated to room temperature. Gradient dilutions of the DENV-NS1 antigen were prepared using 0.1 M PBS buffer (pH 7.4) and the co-reactant as diluents. During the condition optimization phase, tripropylamine (TPA) was used as the co-reactant, and the antigen was diluted to a working concentration of 20,000 pg/mL with PBS buffer and TPA at a volume ratio of 1:1 (the co-reactant will be optimized later).

#### 2.2.4 Optimization of the co-reactant in the electrochemiluminescence immunoassay system

To identify the optimal co-reactant type, this study compared three groups: the Roche Elecsys Buffer group, the TPA group, and the PBS blank group.

On the basis of determining the co-reactant type, the mixing ratio with the sample was further optimized. Groups with sample-to-co-reactant volume ratios of 3:1, 2:1, and 1:1 were set up, and the solutions were thoroughly mixed before sample loading.

After assembling the complete test strip, 100 μL of DENV-NS1 antigen sample was slowly dropped onto the sample pad and allowed to undergo chromatographic separation in the dark for 15 min. Following chromatography, the detection chip was inserted into a BPCL three-channel electrochemiluminescence detector, and cyclic voltammetry was performed with a scanning potential of 0.2–1.5 V and a photomultiplier tube voltage of 900 V for ECL detection, with results obtainable within 30 s. Each group was tested in triplicate to ensure statistical reliability. By comparing the differences in average ECL intensity among the groups, the co-reactant group yielding the highest average ECL intensity was selected, and the optimal co-reactant type and its mixing ratio with the sample were determined.

#### 2.2.5 Optimization of the detection pad material

In this study, four types of paper-based materials were selected for parallel comparison and screening: cellulose chromatography paper Whatman NO. 1, nitrocellulose microporous membrane CN95, nitrocellulose microporous membrane Whatman AE 100, and nitrocellulose microporous membrane PT-0.45 μm. By systematically evaluating the ECL intensity of the four materials under the same experimental conditions, the most suitable material was selected as the detection pad for the ECL immunochromatographic test strip developed in this study, providing an optimal solid-phase carrier for subsequent experiments.

Different detection pad length groups were set, namely, 10 mm, 15 mm, and 20 mm. Complete test strips were assembled using detection pads of each length, and 100 μL of DENV-NS1 antigen sample was slowly added dropwise to the sample pad. Chromatography was performed in the dark for 15 min, followed by ECL detection. Three replicates were set for each length group. By comparing the differences in average ECL signal intensity among the three groups, the detection pad length yielding the highest average ECL signal intensity was selected as the standardized parameter for subsequent experiments.

#### 2.2.6 Optimization of the concentration ratio of labeled antibody to capture antibody

In this study, the concentration ratio of the labeled antibody to the capture antibody was optimized. Four groups with different concentration ratios of labeled antibody to capture antibody—1:1, 1:2, 1:3, and 1:4—were set up for parallel comparison. Specifically, the labeled antibody concentration was fixed at 1 mg/mL, while the spray-coating concentrations of the capture antibody on the detection pad were adjusted to 1 mg/mL, 2 mg/mL, 3 mg/mL, and 4 mg/mL, respectively.

After assembling the complete test strips, 100 μL of the DENV-NS1 antigen sample was slowly dropped onto the sample pad and allowed to undergo chromatography for 15 min in the dark. Following chromatography, ECL detection was performed using a BPCL three-channel electrochemiluminescence detector. Three replicates were set for each group. By comparing the differences in the average ECL intensity among the groups, the group yielding the highest average ECL intensity was selected to determine the optimal concentration ratio of the labeled antibody to the capture antibody.

#### 2.2.7 Optimization of chromatographic time

Using the optimized conditions, including the material and length of the detection pad, the concentration ratio of labeled antibody to capture antibody, and the type and addition ratio of the co-reactant, a single batch of test strips was prepared to ensure consistency. A 100 μL sample of DENV-NS1 antigen was slowly dropped onto the sample pad, and the test strips were allowed to develop under light-protected conditions for 5, 7, 10, 12, and 15 min, respectively. After development, electrochemiluminescence detection was performed using a BPCL three-channel electrochemiluminescence detector. Three replicates were set for each group, and the average ECL intensity was compared among groups to identify the group with the highest average ECL intensity and determine the optimal development time.

#### 2.2.8 Performance evaluation of the dengue virus ECLIA test strip

##### 2.2.8.1 Establishment of the standard curve

Using the same sample dilution method as described in the previous experiment, DENV-NS1 antigen was diluted to the following concentrations: 20,000 pg/mL, 4,000 pg/mL, 800 pg/mL, 160 pg/mL, and 32 pg/mL, and kept on ice until use. DENV electrochemiluminescence immunochromatographic test strips from the same batch were taken, and 100 μL of each concentration of DENV-NS1 sample was pipetted onto the sample pad of the test strip. Three replicates were set for each concentration. The reaction was carried out under light-protected conditions according to the optimized chromatographic time, followed by ECL detection, and the ECL intensity corresponding to each concentration was recorded.

To achieve a better linear fit, logarithmic transformation was applied to the concentration data. The logarithm of DENV-NS1 concentration (lg C_DENV_) was used as the horizontal coordinate, and the corresponding electrochemiluminescence intensity (I_ECL_) was used as the vertical coordinate. The data were imported into Origin 2018 statistical analysis software for processing and analysis to establish a linear regression equation between I_ECL_ and lg C_DENV_, and the correlation coefficient (*R* ^*2*^) of the regression equation was calculated to evaluate the goodness of fit of the model. An *R* ^*2*^ value closer to 1 indicates a stronger correlation and better predictive capability of the model.

##### 2.2.8.2 Determination of Limit of Detection (LOD)

In an electrochemiluminescence immunoassay system, the LOD is typically determined based on the statistical characteristics of the signal distribution of blank samples, combined with back-calculation using a standard curve.

In accordance with the guidelines of the International Union of Pure and Applied Chemistry (IUPAC) and relevant bioanalytical method validation guidelines, the signal intensity generated by a test sample must be at least three times the standard deviation of the background noise of the blank to be considered a positive signal. Based on the electrochemiluminescence signal intensity data obtained from 10 blank samples, the mean (Mean_blank_) and standard deviation (SD_blank_) were calculated. According to the criterion of signal-to-noise ratio (S/N) = 3, the signal threshold corresponding to the theoretical LOD was calculated using the formula: I_LOD_ = Mean_blank_ + 3 × SD_blank_.

Ten blank samples with a DENV-NS1 concentration of 0 pg/mL were prepared. From the same batch of prepared DENV electrochemiluminescence immunochromatographic test strips, 100 μL of the blank sample solution was added to the sample pad of each of the 10 strips. The strips were processed under the optimized detection conditions, and ECL detection was performed. The ECL intensity of each blank sample was recorded, and the Mean_blank_, SD_blank_, and I_LOD_ were calculated.

The luminescence signal intensity corresponding to the obtained theoretical LOD was then substituted into the established standard curve regression equation for back-calculation to determine the minimum detectable concentration of DENV-NS1 by the test strip.

##### 2.2.8.3 Specificity analysis

To verify the specificity of the electrochemiluminescence immunochromatographic test strip constructed in this study for detecting dengue virus NS1 protein, a series of experimental groups were set up: dengue virus (DENV) group, Japanese encephalitis virus (JEV) group, Zika virus (ZIKV) group, and PBS blank group. The sample preparation method for each group was the same as that described in section 1.2.3 for DENV-NS1 sample preparation.

A batch of DENV electrochemiluminescence immunochromatographic test strips prepared under the previously optimized conditions was taken. 100 μL of each of the four groups of samples was slowly dropped onto the sample pads of the corresponding test strips. The reaction was carried out under light-protected conditions for the optimized chromatography time, followed by ECL detection. By comparing the differences in the average ECL intensity among the groups, it was determined whether the electrochemiluminescence immunochromatographic test strip exhibited cross-reactivity with other viruses.

##### 2.2.8.4 Intra-assay repeatability analysis

To verify the intra-assay repeatability of the DENV electrochemiluminescence immunochromatographic test strip developed in this study, sample pads, conjugate pads, detection pads, and absorbent pads from the same batch were used to assemble and prepare 10 test strips. Consistency of all operational conditions was strictly controlled throughout the experiment.

Using the same sample dilution method as described in previous experiments, the DENV-NS1 antigen was diluted to a working solution of 20,000 pg/mL. The 10 test strips prepared from the same batch were each loaded with 100 μL of the sample solution, which was slowly and evenly added dropwise to the sample pad of each strip. Chromatography was performed under light-protected conditions according to the optimized detection time, followed by ECL measurement. The ECL intensity corresponding to each test strip was recorded, and the mean, standard deviation (SD), and coefficient of variation (CV) were calculated.

##### 2.2.8.5 Storage stability analysis

To evaluate the long-term stability of the test strips under room temperature storage conditions, sample pads, conjugate pads, detection pads, and absorbent pads from the same batch were used to assemble and prepare 30 test strips to meet the testing requirements at three time points: day 0, day 30, and day 60 (10 strips per time point). The prepared test strips were placed in aluminum foil bags with an adequate amount of desiccant and sealed for storage.

Using the same sample dilution method as described in previous experiments, the DENV-NS1 antigen was diluted to a working solution of 20,000 pg/mL. At each time point, 10 test strips were taken, and 100 μL of the sample solution was slowly and evenly added dropwise to the sample pad of each strip. The reaction was carried out under light-protected conditions according to the optimized detection time, followed by ECL measurement. The ECL intensity corresponding to each test strip at each time point was recorded, and the mean, SD, and CV were calculated.

##### 2.2.8.6 Inter-assay stability analysis

To verify the inter-assay stability of the DENV electrochemiluminescence immunochromatographic test strip developed in this study, three independent batches were prepared using the same fabrication process, with 10 test strips per batch.

Using the same sample loading and detection method as described in previous experiments, the 10 test strips from each of the three batches were subjected to ECL measurement. The ECL intensity corresponding to each test strip in each batch was recorded, and the mean, SD, and CV were calculated.

##### 2.2.8.7 Methodological validation

In this study, a total of 20 clinical serum samples were collected. Using nucleic acid testing (NAT) results as the gold standard, the detection results obtained by the ECLIA were organized into a four-fold table, and the numbers of true positives (TP), false positives (FP), false negatives (FN), and true negatives (TN) were calculated. Based on the above data, the agreement between the two detection methods was evaluated using the *Kappa* statistic, calculated as *Kappa* = (P_0_ - P_e_)/(1 - P_e_). A *Kappa* value of ≥0.75 indicates excellent agreement, a value between 0.40 and 0.75 indicates moderate-to-substantial agreement and a value ≤0.40 indicates poor agreement.

#### 2.2.9 Statistical analysis

Statistical analysis of the detection results was performed using SPSS 25.0. Comparisons of means between groups were conducted using analysis of variance (ANOVA). If the overall differences were statistically significant, pairwise comparisons were subsequently performed using the t-test. A two-tailed test was employed, and a *p*-value <0.05 was considered statistically significant. In the figures, *p* < 0.05, *p* < 0.01, and *p* < 0.001 are denoted by *, **, and ***, respectively.

For all optimization experiments, three technical replicates per group were used. This sample size was determined based on preliminary data showing a coefficient of variation of approximately 5% for ECL intensity measurements, and was calculated to provide sufficient power to detect a 30% difference between groups using a two-sided t-test with α = 0.05 and β = 0.20. For stability evaluations, ten test strips per batch were tested, exceeding the minimum of six replicates recommended by the CLSI EP05-A3 guideline for quantitative assay precision evaluation.

## 3 Results

### 3.1 Characterization of the ECL bioprobe

**FIGURE 2:**
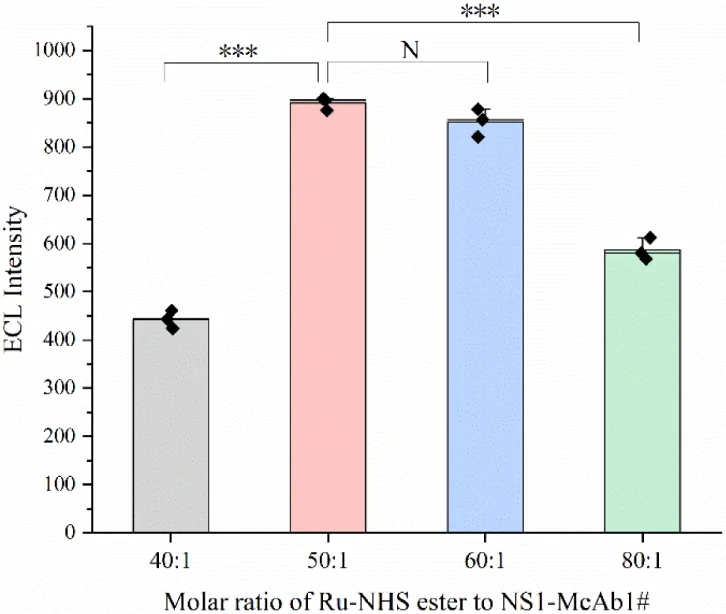
ECL intensity corresponding to different molar ratios of Ru-NHS ester to NS1-McAb1#.

Based on the different molar ratios of Ru-NHS ester to NS1-McAb1# during the preparation of the ruthenium-labeled antibody solution, groups with ratios of 40:1, 50:1, 60:1, and 80:1 were established. The mean ECL intensities of the aforementioned groups were 443, 891, 852, and 587 (*n* = 3), respectively, as shown in Figure 2, with statistically significant differences among them (*p* < 0.001). When the molar ratios of Ru-NHS ester to NS1-McAb1# were set at 50:1 and 60:1, the average ECL intensities were similar, and the difference was not statistically significant (*p* > 0.05). Considering cost-effectiveness, a molar ratio of 50:1 was selected for the preparation of the ruthenium-labeled antibody.

### 3.2 Reaction time for ruthenium-labeled antibody

**FIGURE 3:**
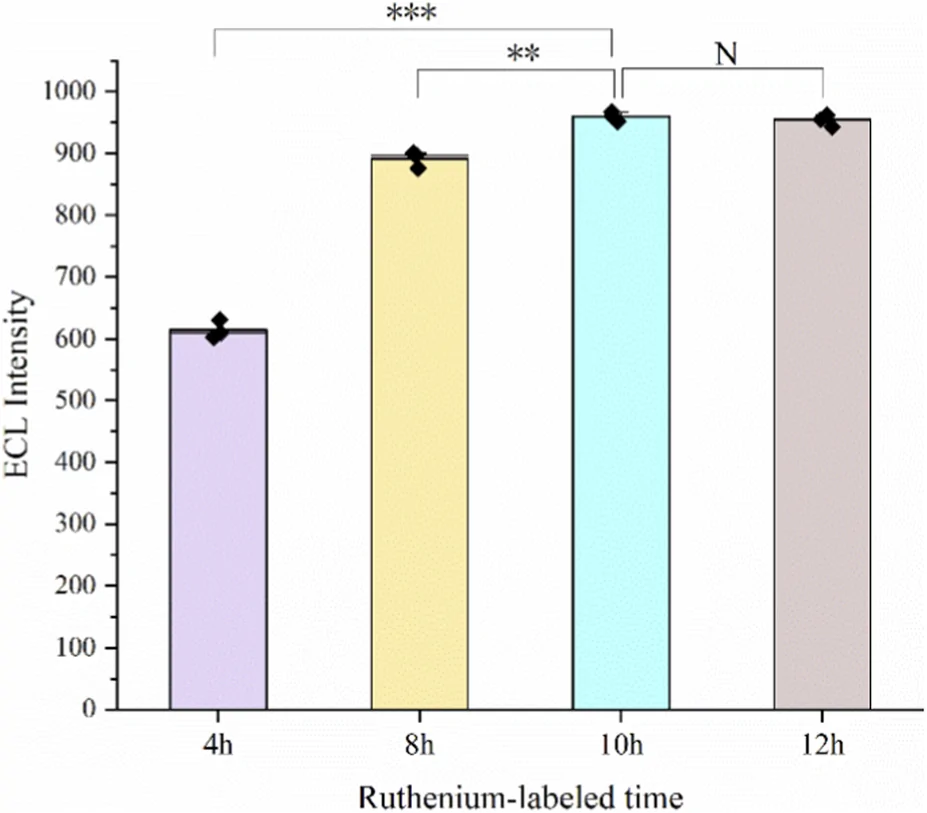
ECL intensity corresponding to different ruthenium labeling times.

Under a molar ratio of 50:1 for Ru-NHS ester to NS1-McAb1#, different reaction time groups were set at 4 h, 8 h, 10 h, and 12 h. The corresponding mean ECL intensities for these groups were 615, 891, 960, and 953 (*n* = 3), respectively, as shown in Figure 3, with statistically significant differences observed (*p* < 0.05). No significant difference in mean ECL intensity was found between the 10 h and 12 h reaction times (*p* > 0.05). Considering time efficiency, 10 h was selected as the optimal reaction time for Ru-NHS ester and NS1-McAb1#.

### 3.3 Optimization of co-reactants for ECL

**FIGURE 4:**
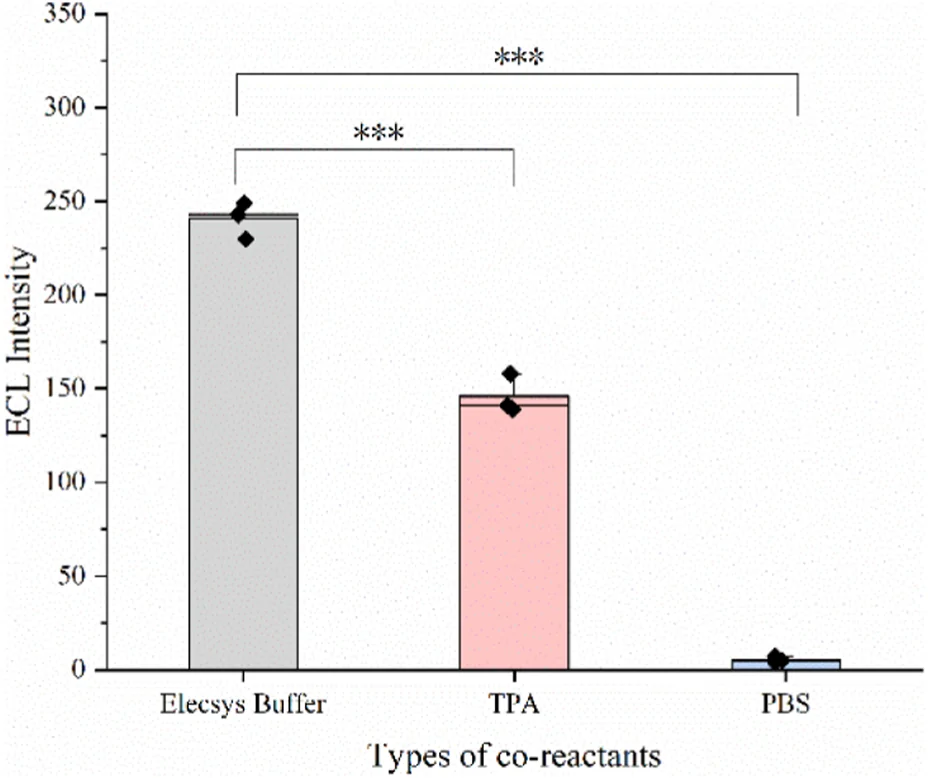
ECL intensity corresponding to different types of co-reactants.

To determine the optimal co-reactant, three groups were set up: the Elecsys Buffer group, the TPA group, and the PBS blank group. A 20,000 pg/ml DENV-NS1 antigen dilution was used as the sample, and the sample was thoroughly mixed with the co-reactant at a 1:1 volume ratio. The mixture was then added dropwise to the detection zone of the electrode sheet for ECL measurement. The average ECL intensities for each group were 241, 146, and 5, respectively (*n* = 3), as shown in Figure 4, with statistically significant differences (*p* < 0.001). Among them, the Elecsys Buffer group exhibited the highest average ECL intensity; therefore, Elecsys Buffer was selected as the co-reactant for this study.

**FIGURE 5:**
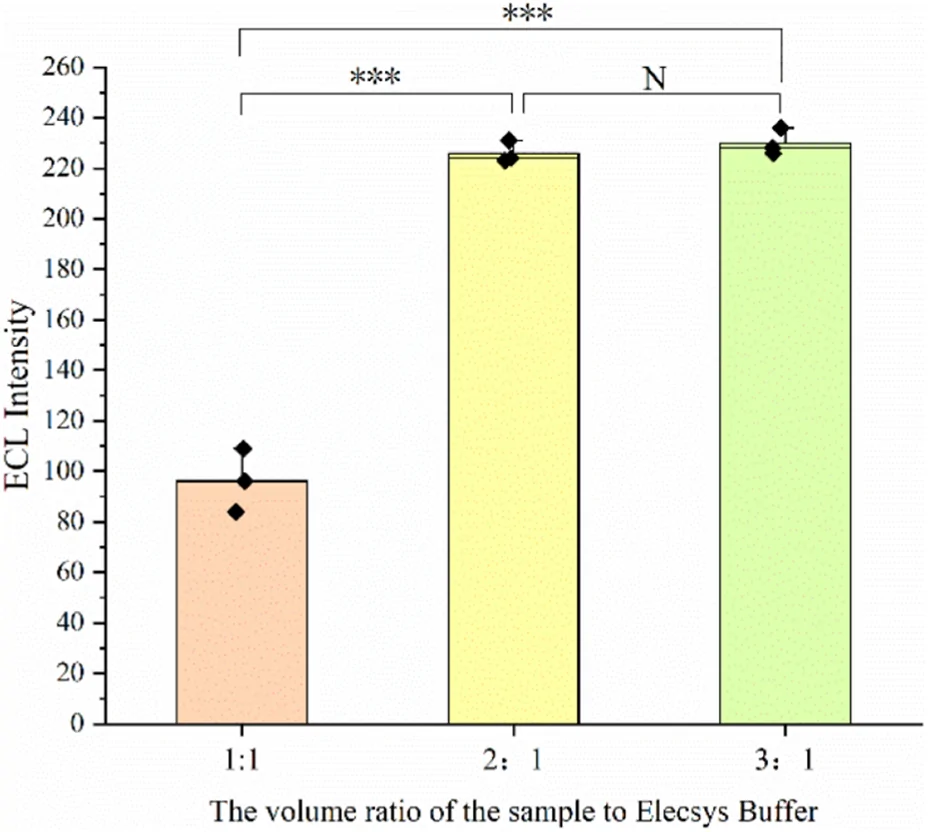
ECL intensity corresponding to different volume ratios of the sample to the co-reactant.

Three groups with sample-to-Elecsys Buffer volume ratios of 1:1, 2:1, and 3:1 were set up. The sample was added dropwise to the sample pad of the preliminarily prepared test strip, and after chromatographic development for a certain period, ECL detection was performed. The average ECL intensities for the above groups were 96, 226, and 230, respectively (*n* = 3), as shown in Figure 5, with a statistically significant difference (*p* < 0.001). When the sample-to-Elecsys Buffer volume ratios were 2:1 and 3:1, the average ECL intensities were similar, and the difference was not statistically significant (*p* > 0.05). Considering cost savings, a sample-to-Elecsys Buffer volume ratio of 2:1 was selected as the addition ratio.

### 3.4 Optimization of paper-based materials

**FIGURE 6:**
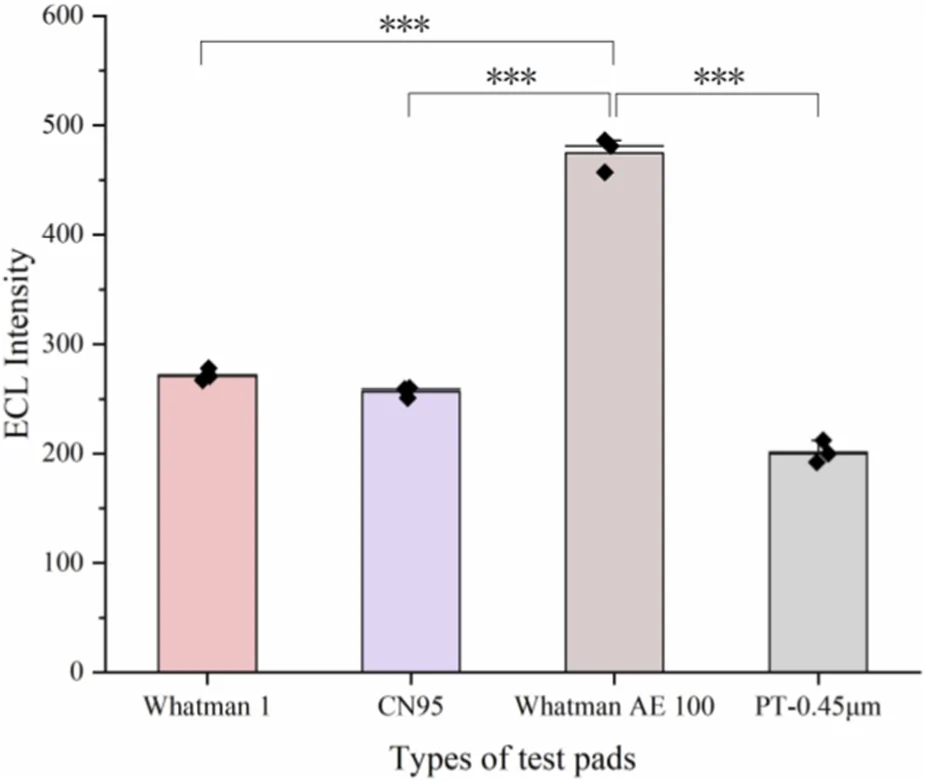
ECL intensity corresponding to different detection pad materials.

In this study, Whatman NO. 1 filter paper, CN95 membrane, Whatman AE 100 membrane, and PT-0.45 μm were evaluated for selection. The treated sample pads, conjugate pads, and absorbent pads were assembled with each of the above materials serving as the detection pad, followed by sample loading and ECL detection. The average ECL intensities for each group were 272, 257, 475, and 201, respectively (*n* = 3), as shown in Figure 6, with a statistically significant difference (*p* < 0.001). The Whatman AE 100 membrane group exhibited the highest average ECL intensity; therefore, Whatman AE 100 membrane was selected as the detection pad for this study.

**FIGURE 7:**
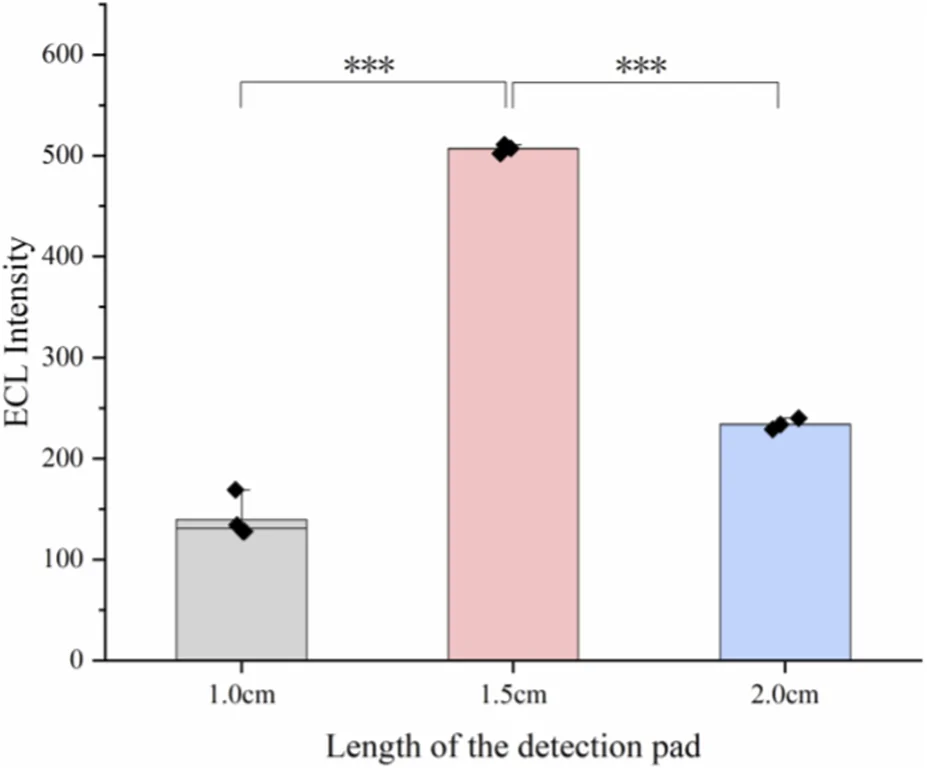
ECL intensity corresponding to different detection pad lengths.

The detection pad lengths were set to 10 mm, 15 mm, and 20 mm, and samples were loaded for ECL detection. The average ECL intensities for each group were 144, 507, and 234, respectively (*n* = 3), as shown in Figure 7, with statistically significant differences (*p* < 0.001). The group with a detection pad length of 15 mm exhibited the highest average ECL intensity; therefore, the detection pad length was set to 15 mm.

### 3.5 Ratio of labeled antibody to capture antibody and concentration optimization

**FIGURE 8:**
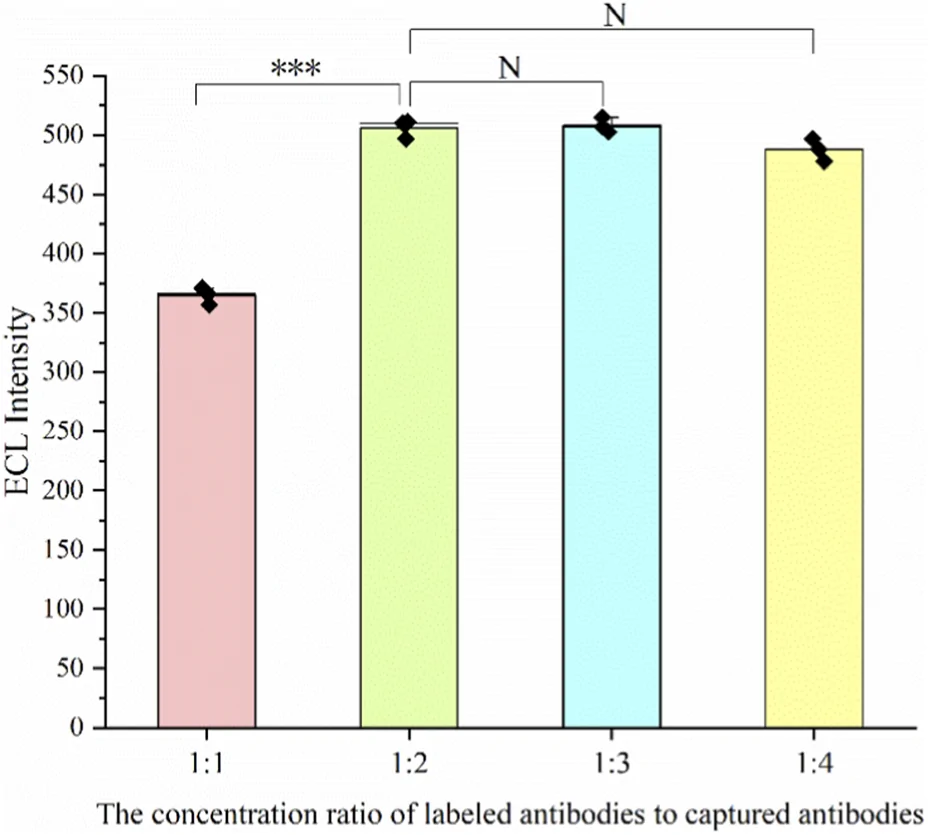
ECL intensity corresponding to different concentration ratios of labeled antibody to capture antibody.

In this study, ratios of 1:1, 1:2, 1:3, and 1:4 (labeled antibody to capture antibody) were tested. After sample loading and ECL detection, the mean ECL intensities for each group were 365, 506, 508, and 488 (*n* = 3), respectively, as shown in Figure 8. When the ratios were set at 1:2, 1:3, and 1:4, the average ECL intensities were significantly higher and comparable to each other, with no statistically significant differences observed (*p* > 0.05). Considering cost-effectiveness, a ratio of 1:2 was selected for subsequent experiments, corresponding to concentrations of 1 mg/mL for the labeled antibody and 2 mg/mL for the capture antibody.

### 3.6 Optimization of detection time

**FIGURE 9:**
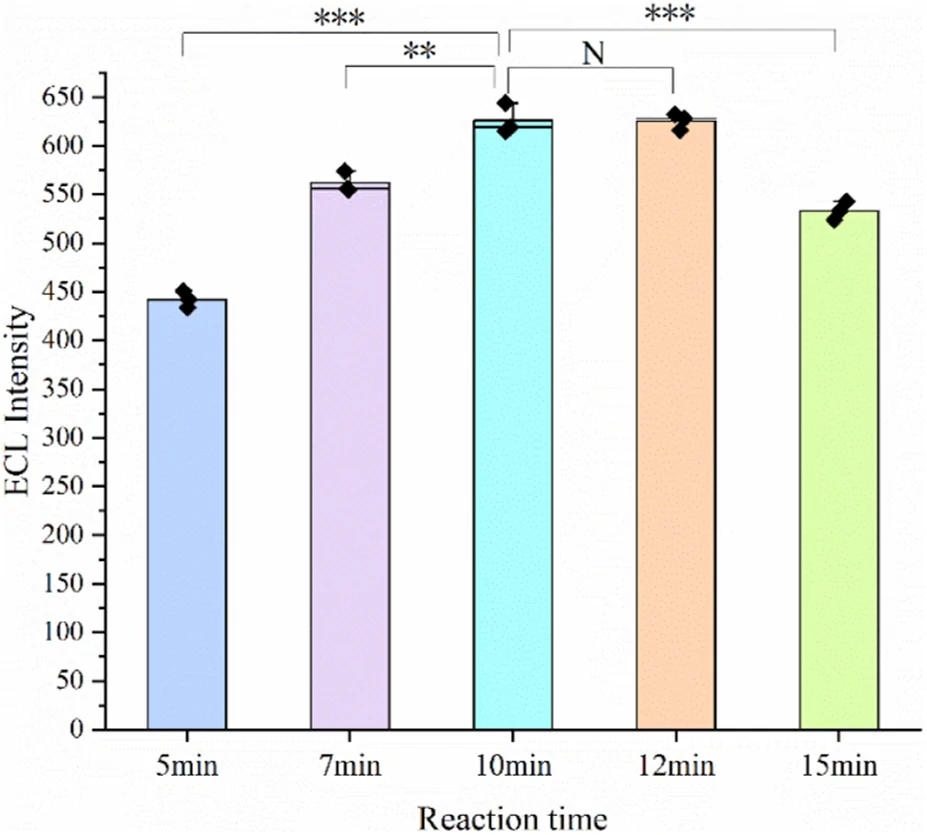
ECL intensity corresponding to different reaction times.

In this study, different reaction time groups were set at 5 min, 7 min, 10 min, 12 min, and 15 min. After sample addition and ECL measurement, the average ECL intensities for each group were 442, 561, 626, 625, and 524 (*n* = 3), respectively, as shown in Figure 9. Both the 10 min and 12 min reaction times yielded relatively high average ECL intensities, which were comparable and showed no statistically significant difference (*p* > 0.05). Considering time efficiency, 10 min was selected as the detection time for this study.

### 3.7 Performance analysis of the DENV electrochemiluminescence immunochromatographic test strips

**FIGURE 10:**
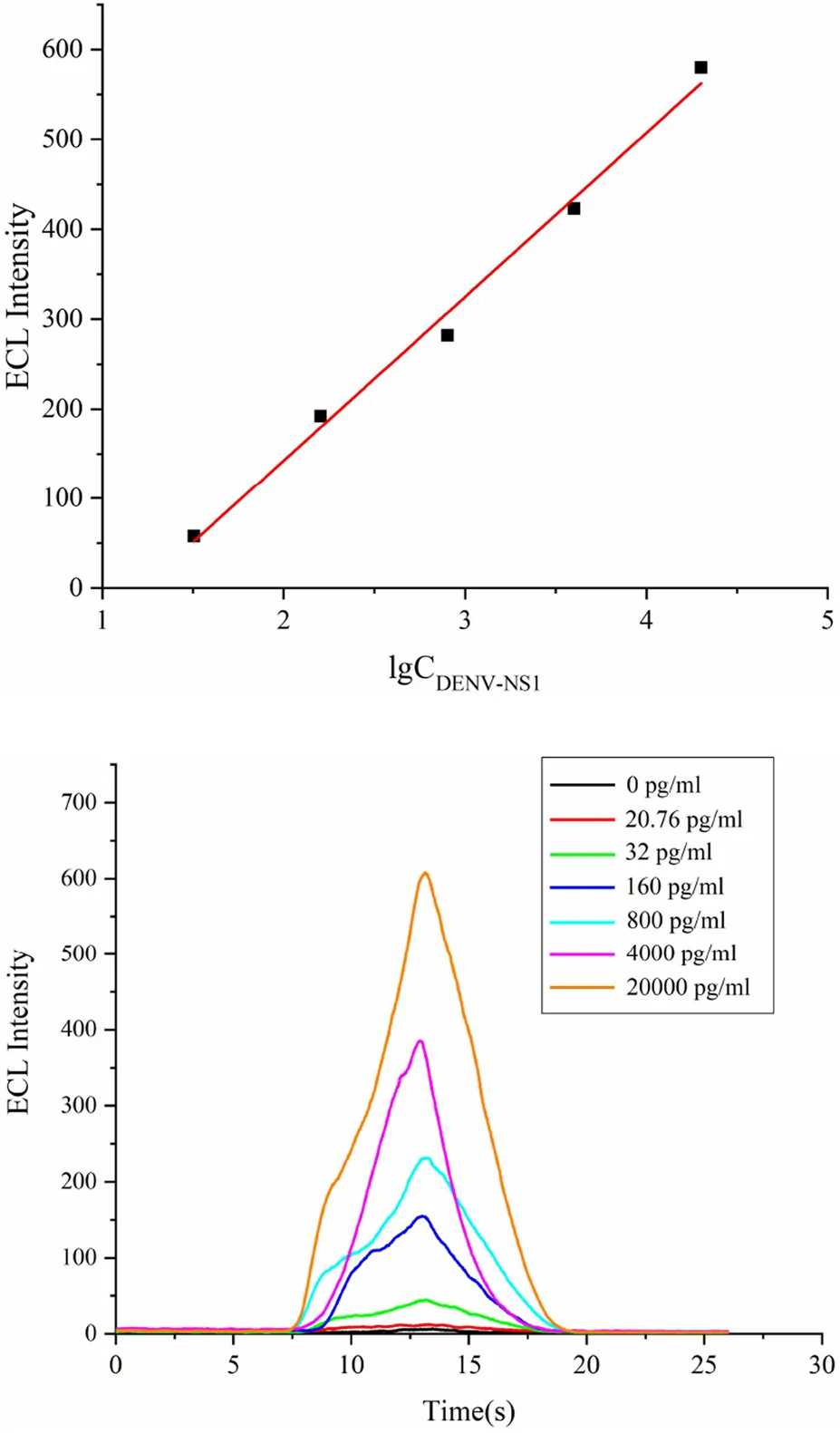
ECL intensity corresponding to different concentrations of DENV-NS1 and their linear relationship.

The relationship between viral load and the ECL intensity of the test strip was analyzed. The linear regression equation was I_ECL_ = 182.41lgC_DENV-NS1_- 222.55, with a correlation coefficient *R* ^*2*^ = 0.9922 (*p* < 0.001), indicating a good correlation and a positive correlation. DENV-NS1 at concentrations of 0, 20.76, 32, 160, 800, 4,000, and 20,000 pg/mL were detected, and the results are shown in Figure 10.

### 3.8 Repeatability and stability analysis of the DENV electrochemiluminescence immunochromatographic test strips

**FIGURE 11:**
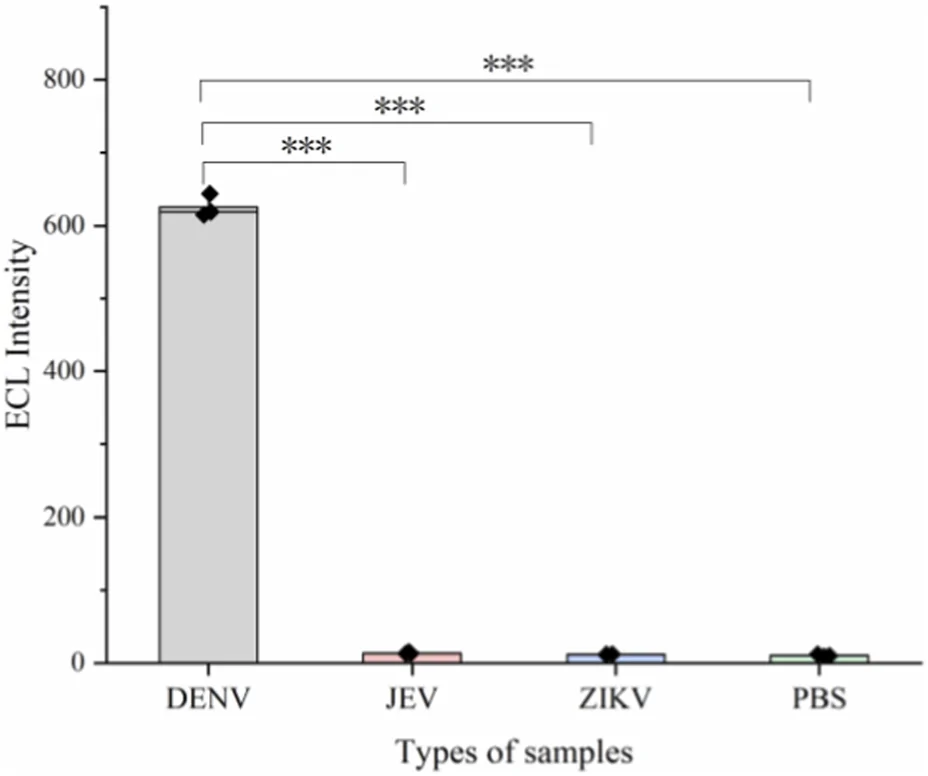
ECL intensity corresponding to different antigen types.

In this study, the DENV, JEV, ZIKV groups and the PBS blank group were set up. The experimental results showed that the average ECL intensity of the DENV-positive control group was significantly higher than that of the other groups, and the difference was statistically significant (*p* < 0.001), as shown in Figure 11. This indicates that the DENV electrochemiluminescence immunochromatographic test strip developed in this study exhibits high specificity for the DENV antigen, with no significant cross-reactivity with JEV, ZIKV, or other background matrices.

### 3.9 Repeatability and stability analysis of the DENV electrochemiluminescence immunochromatographic test strips

To evaluate the repeatability of the DENV electrochemiluminescence immunochromatographic test strip assay, ten identical strips were used to detect the same batch of sample containing DENV NS1 at a concentration of 20,000 pg/mL via ECL measurement. As shown in Table 1, the mean ECL intensity obtained from the ten replicate tests was 645, with a standard deviation (SD) of 26 and a coefficient of variation (CV) of 4%.

**TABLE 1:** Repeatability of ECL detection using test strips.

Concentration (pg/mL)	Mean ECL intensity (*n* = 10)	SD	CV
20,000	645	26	4%

To evaluate the storage stability of the DENV electrochemiluminescence immunochromatographic test strip, test strips from the same batch were used to detect DENV NS1 at a concentration of 20,000 pg/mL at three time points (day 0, day 30, and day 60), with 10 test strips per time point. The results are shown in Table 2. The mean ECL intensities at each time point were 645, 655, and 646, respectively. The overall mean ECL intensity across all time points was 649, with a standard deviation of 5.41 and a coefficient of variation of 0.83%.

**TABLE 2:** Storage stability of the Test Strip in ECL Detection.

Concentration (pg/mL)	Mean ECL intensity at each time point (*n* = 10)	Average ECL intensity at each time point (M)	SD	CV
20,000	645 655 646	649	5.41	0.83%

To evaluate the inter-assay stability of the DENV electrochemiluminescence immunochromatographic test strip, three batches of test strips were used to detect DENV-NS1 at a concentration of 20,000 pg/mL by ECL, with 10 strips per batch. The results are shown in Table 3. The mean ECL intensities of the test strips in each batch were 645, 651, and 656, respectively. The overall mean of the average ECL intensities across batches was 651, with a standard deviation of 5.25 and a coefficient of variation of 0.81%.

**TABLE 3:** Inter-assay stability of the test strip for ECL detection.

Concentration (pg/mL)	Mean ECL intensity for each batch (n = 10)	Average ECL intensity for each batch (M)	SD	CV
20,000	645 651 656	651	5.25	0.81%

### 3.10 Methodological validation

In this study, the DENV electrochemiluminescence immunochromatographic strip was used to detect serum specimens from 20 dengue-suspected subjects. The results are shown in Table 4. The calculated *Kappa* value was 0.625 (*p* < 0.05). According to the grading criteria, a *Kappa* value between 0.40 and 0.75 indicates substantial agreement, demonstrating that the electrochemiluminescence immunochromatographic strip developed in this study is in moderate-to-substantial agreement with nucleic acid testing results and yields reliable detection outcomes.

**TABLE 4:** Methodological validation of the test strip.

ECLIA	RT- PCR		Total
	Patient	Non-patient	
Positive	4	2	6
Negative	1	13	14
Total	5	15	20

## 4 Discussion

The ECLIA method established in this study requires only a 10-min reaction after sample loading before it can be subjected to ECL detection, with results read within 30 s, thereby meeting the requirements for rapid detection.

The ECLIA developed in this study achieved a minimum detection limit of 20.76 pg/mL. Notably, this value is approximately 1–2 orders of magnitude lower than the detection limits of commercial DENV NS1 antigen detection kits, which typically range from 0.5 to 5 ng/mL for products from brands such as Wondfo Bio, Abon Bio, and Abbott (Zhang, 2015). The proposed method also compares favorably with the multilayer fluorescence immunoassay reported by Browne (Browne et al., 2025) (351 pg/mL) and the immunosensor reported by Hasan (Hasan et al., 2024) (100 ng/mL). This substantial improvement in analytical sensitivity suggests that the proposed ECLIA has the potential to detect DENV infection at earlier stages or in samples with low viral loads. A strong positive correlation was observed between DENV concentration and the ECL intensity of the test strip. Within a certain concentration range, the antigen concentration can be inferred from the ECL intensity, enabling quantitative detection. However, this method has a limitation: when the antigen concentration is excessively high, far exceeding the antibody concentration, excessive antigen competes for the binding sites of the capture antibody, resulting in a decrease in the formation of double-antibody sandwich complexes. Consequently, the detectable complexes are reduced, leading to a decline in the electrochemiluminescence intensity signal. Future studies will aim to address this limitation.

DENV, ZIKV, and JEV share the same transmission routes and exhibit similar clinical symptoms. Cross-infection among these three viruses may lead to false positives in DENV detection. In this study, commercially available antigens were used as samples for comparative detection, and the results demonstrated that the newly established method exhibited good specificity. However, it is important to acknowledge several limitations in the current specificity and anti-interference validation. The specificity assessment was performed using commercially available antigens rather than authentic clinical samples. While purified antigens allow for controlled and reproducible evaluation of cross-reactivity between viral proteins (Voyvodic et al., 2022), they do not fully recapitulate the complex matrix of real biological specimens (e.g., serum, plasma, or whole blood), which may contain various interfering substances (Adil and Shamsi, 2025). Despite the anti-interference strategies incorporated into the test strip, such as HBR pretreatment, these matrix components could still potentially affect its specificity and reliability in real-world diagnostic settings. Therefore, the specificity and anti-interference capability of this DENV electrochemiluminescence immunochromatographic test strip require further validation using authentic clinical samples. Future studies will involve the collection of real clinical samples to comprehensively evaluate both the analytical specificity and the practical anti-interference performance (matrix effects from complex biological specimens). Such validation will be essential for translating this proof-of-concept technology into a clinically applicable point-of-care testing tool.

According to the standard specified in the national standard GB/T 26,124–2011 Clinical chemistry *in vitro* diagnostic reagents (kits) (National Technical Committee on Medical Clinical Laboratory and In Vitro Diagnostic Systems, 2011), the coefficient of variation for intra-assay repeatability is required to be less than 5.0%. In this experiment, the coefficient of variation of 4.0% was below this standard, indicating that the DENV electrochemiluminescence immunochromatographic test strip developed in this study exhibits good intra-assay repeatability. For inter-assay stability, the required coefficient of variation is less than 10.0% according to the same standard; the coefficient of variation of 0.81% obtained in this experiment was below this threshold, demonstrating good inter-assay stability of the test strip. Regarding long-term stability under room temperature storage conditions, according to the industry standard YY/T 1579–2018 *In vitro* diagnostic medical devices—Evaluation of stability of *in vitro* diagnostic reagents (National Technical Committee on Medical Clinical Laboratory and In Vitro Diagnostic Systems SAC/TC 136, 2018), the coefficient of variation is required to be within 10%. In this experiment, the coefficient of variation of 0.83% fell below this criterion, indicating that the test strip possesses good storage stability. However, the long-term storage stability of this method still requires further investigation.

In the methodological validation of the DENV electrochemiluminescence immunochromatographic strip, the ECLIA results showed moderate-to-substantial agreement with NAT (*Kappa* = 0.625), preliminarily confirming the feasibility of this strip for clinical sample detection. However, certain limitations should be acknowledged: the clinical sample size was relatively small, which limited the statistical power. This methodological evaluation represents a proof-of-concept study that only provides initial evidence for the technical feasibility of the newly developed method; its generalizability and broader applicability remain to be further validated. Future studies will expand the sample size to ensure that the statistical power meets the requirements for methodological validation.

In addition, the electrode sheets and paper materials selected in this study are low in cost, meeting the application requirements for low-cost detection.

In summary, the ECLIA established in this study does not require large-scale instruments and enables low-cost, rapid quantitative detection of DENV, with good sensitivity, specificity, and stability under the optimized laboratory conditions. These results suggest that the proposed method holds promise as a candidate for on-site rapid testing; however, this claim remains to be validated in real-world clinical settings. In addition, the present work provides a useful methodological reference for the development of similar paper-based electrochemiluminescence assays for other infectious disease biomarkers.

## 5 Conclusion

The dengue virus electrochemiluminescence immunochromatographic test strip developed in this study consists of a paper-based channel composed of a sample pad (RB-45), a conjugate pad (RB-45), a detection pad (Whatman AE 100), and an absorbent pad (ABP-S270). The paper-based channel is fixed onto a screen-printed electrode sheet, with the detection pad placed over the detection zone of the electrode. The monoclonal antibody NS1-McAb1# labeled with Ru-NHS ester serves as the labeled antibody and is immobilized on the conjugate pad as a biological probe, while the capture antibody DN-REAB-G2-006 is immobilized on the detection pad. Importantly, beyond the basic structural design, special attention was paid to mitigating matrix interference. To address this issue, the present study incorporated multiple anti-interference strategies: (1) the sample pad and conjugate pad was pretreated with HBR to neutralize potential interfering antibodies; (2) Elecsys buffer was selected as the optimal co-reactant to minimize non-specific binding.

After the antigen dilution buffer is added to the sample pad, a 10-min chromatographic reaction is performed under light-protected conditions. Driven by capillary action within the paper-based channel, the antigen migrates to the conjugate pad, where it specifically binds to the labeled antibody to form an “antigen–antibody complex.' The complex continues to migrate to the detection pad and is captured by the capture antibody, forming a double-antibody sandwich complex of “labeled antibody–antigen–capture antibody.' The electrode sheet is then inserted into a three-channel electrochemiluminescence detector, and cyclic voltammetry scanning is performed in the detection zone at a scan rate of 0.1 V/s for electrochemiluminescence detection, with results read within 30 s.

This detection method exhibits excellent analytical performance in terms of quantitative detection, sensitivity, specificity, reproducibility, and operational simplicity under controlled laboratory conditions. These findings suggest that the test strip has the potential to become a useful tool for on-site rapid screening and viral load monitoring. Furthermore, the methodology developed here may serve as a technical reference for the design of similar paper-based biosensors for other pathogen detection applications.

## Data Availability

The raw data supporting the conclusions of this article will be made available by the authors, without undue reservation.
